# Design, Synthesis and Tumour-Selective Toxicity of Novel 1-[3-{3,5-Bis(benzylidene)-4-oxo-1-piperidino}-3-oxopropyl]-4-piperidone Oximes and Related Quaternary Ammonium Salts

**DOI:** 10.3390/molecules26237132

**Published:** 2021-11-25

**Authors:** Praveen K. Roayapalley, Jonathan R. Dimmock, Lisett Contreras, Karol S. Balderrama, Renato J. Aguilera, Hiroshi Sakagami, Shigeru Amano, Rajendra K. Sharma, Umashankar Das

**Affiliations:** 1 Drug Discovery and Development Research Cluster, University of Saskatchewan, Saskatoon, SK S7N 5E5, Canada; jr.dimmock@usask.ca (J.R.D.); umashankar.usask@gmail.com (U.D.); 2 Department of Biological Sciences and Border Biomedical Research Center, The University of Texas at El Paso, El Paso, TX 79968-0519, USA; lcontreras4@miners.utep.edu (L.C.); karolsbalderrama@gmail.com (K.S.B.); raguilera@utep.edu (R.J.A.); 3 Research Institute of Odontology, Meikai University, Sakado, Saitama 350-0283, Japan; sakagami@dent.meikai.ac.jp (H.S.) shigerua@dent.maikai.a.jp (S.A.); 4 Department of Pathology and Laboratory Medicine, College of Medicine, University of Saskatchewan, Saskatoon, SK S7N 5E5, Canada; rajendra.sharma@usask.ca

**Keywords:** cytotoxins, mitochondrial membrane potential, QSAR, conjugated unsaturated ketones, oximes, quaternary ammonium salts

## Abstract

A novel series of 1-[3-{3,5-bis(benzylidene)-4-oxo-1-piperidino}-3-oxopropyl]-4-piperidone oximes **3a**–**h** and related quaternary ammonium salts **4a**–**h** were prepared as candidate antineoplastic agents. Evaluation against neoplastic Ca9-22, HSC-2 and HSC-4 cells revealed the compounds in series **3** and **4** to be potent cytotoxins with submicromolar CC_50_ values in virtually all cases. In contrast, the compounds were less cytocidal towards HGF, HPLF and HPC non-malignant cells revealing their tumour-selective toxicity. Quantitative structure–activity relationships revealed that, in general, both cytotoxic potency and selectivity index figures increased as the magnitude of the Hammett sigma values rose. In addition, **3a**–**h** are cytotoxic towards a number of leukemic and colon cancer cells. **4b**,**c** lowered the mitochondrial membrane potential in CEM cells, and **4d** induced transient G2/M accumulation in Ca9-22 cells. Five compounds, namely **3c**,**d** and **4c–e**, were identified as lead molecules that have drug-like properties.

## 1. Introduction

The principal objective of this laboratory is the discovery of novel antineoplastic agents. These compounds are conjugated unsaturated ketones. The reasons for pursuing these compounds include the following considerations. First, they are generally reactive towards thiols but less so, if at all, with amino and hydroxyl groups [1,2,3]. Thus, the problem of genotoxicity may be absent with these compounds. Second, by creating conjugated dienones, various series of compounds were formed that have marked cytotoxic potencies [4]. Third, a number of previous studies revealed that conjugated dienones have different modes of action such as increasing the concentration of reactive oxygen species [5] and proteasome inhibition [6]. Thus, many of these conjugated dienones may be referred to as multifunctional ligands. In the present investigation, the conjugated dienone group was mounted on a piperidyl ring to produce a novel series of candidate cytotoxins.

One of the differences between certain neoplasms and various non-malignant cells is the magnitude of the mitochondrial membrane potential (MMP). The MMP in normal cells is approximately −100 to −160 mV and an average value is −139 mV [7,8,9]. On the other hand, the MMP in certain tumours is in excess of −200 mV [10,11]. Thus positively charged compounds may preferentially accumulate in the mitochondria of cancer cells and therefore the design of compounds that have a cytotoxic pharmacophore as well as a positive charge may lead to antineoplastics demonstrating tumour-selective toxicity.

**Figure 1:**
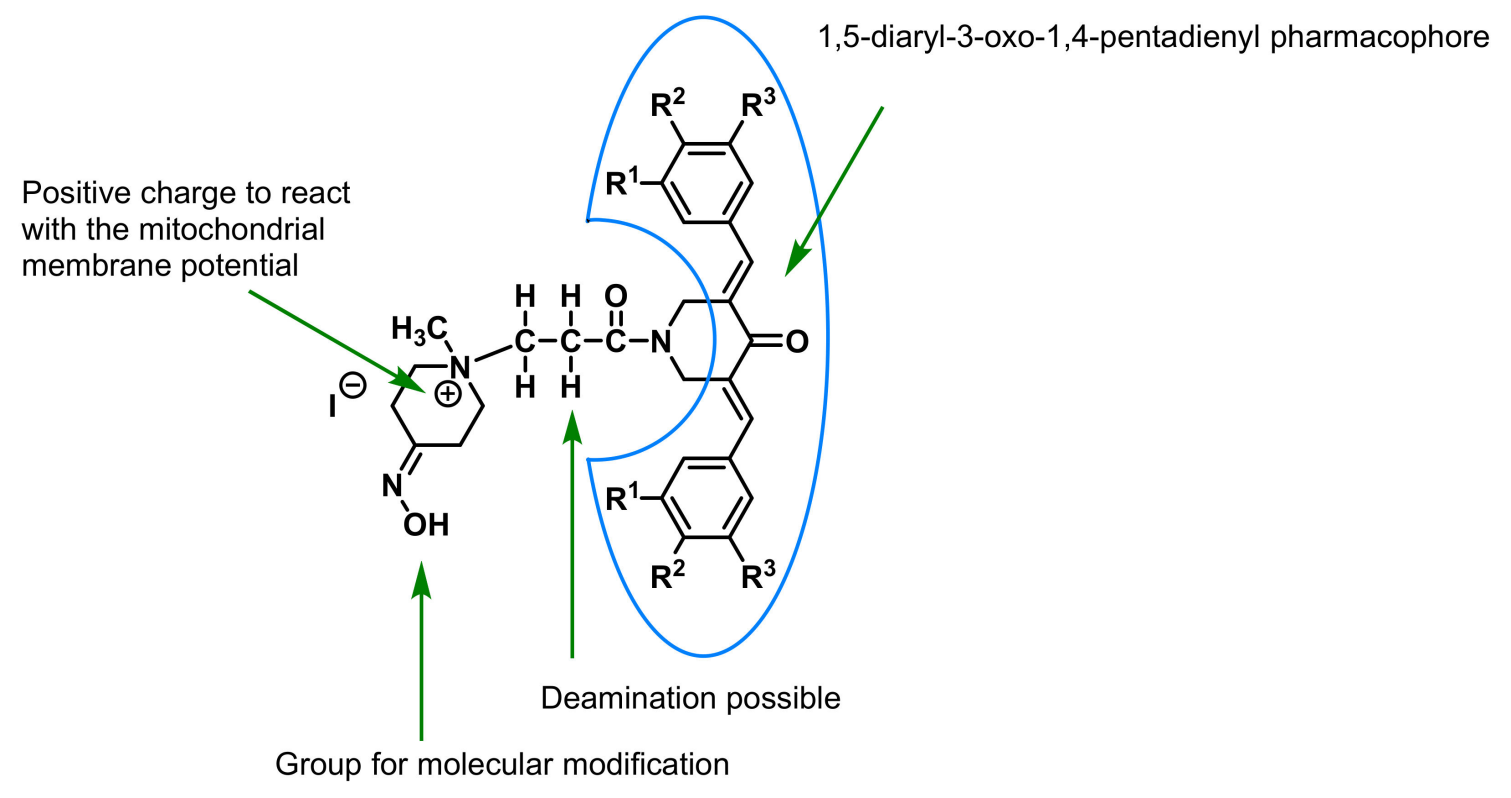
Design of the compounds in series **4**.

Various studies from this laboratory revealed the cytotoxic properties of a number of 1,5-diaryl-3-oxo-1,4-pentadienes that were mounted on heterocyclic and cycloaliphatic scaffolds [12,13]. Hence, this group was incorporated into the design of the target compounds. The choice of aryl substituents was made such that they are found in all four quadrants of a Craig plot for para substituents [14]. Thus, the Hammett σ and Hansch π values for the aryl substituents are +, +(**b**,**c**), +, −(**e**), +(**f**) and −(**g**,**i**). In this way, possible estimates may be made of the contribution of the electronic and hydrophobic properties of the aryl substituents to cytotoxic potencies. In the case of **3h** and **4h**, the 3,4,5-trimethoxyphenyl group is present in a number of potent cytotoxins such as colchicine and combretastatin A-4 [15]. A second feature of the proposed molecules is the presence of a basic group that is partially ionized at physiological pH and a further series of analogs containing a fully charged quadrivalent nitrogen atom. If the effect on the MMP is a dominant one, then the hypothesis is that the quaternary ammonium compounds will show greater cytotoxic potencies than the corresponding amines. A third feature of the target molecules is to display sequential cytotoxicity [16]. This concept is based on the observation that in certain cases after an initial toxic effect from an anticancer agent has occurred, some tumours are now more susceptible to further chemical insult than non-malignant cells [17,18]. Thus, the compounds proposed allow the possibility of deamination to occur leading to the formation of a chemically reactive *N*-acryloyl group, which has the potential to react readily with various cellular constituents. The fourth feature of these compounds is the presence of a hydroxyl group. If the compounds in series **3** and **4** display promising preclinical features, structural modifications can take place at the hydroxyl group such as prodrug formation. In addition, should one or more compounds warrant incorporation into an antibody–drug conjugate, then attachment of the hydroxyl group to a linker can occur. These considerations are illustrated in Figure 1 for the proposed quaternary ammonium compounds **4**.

## 2. Results

**Scheme 1:**
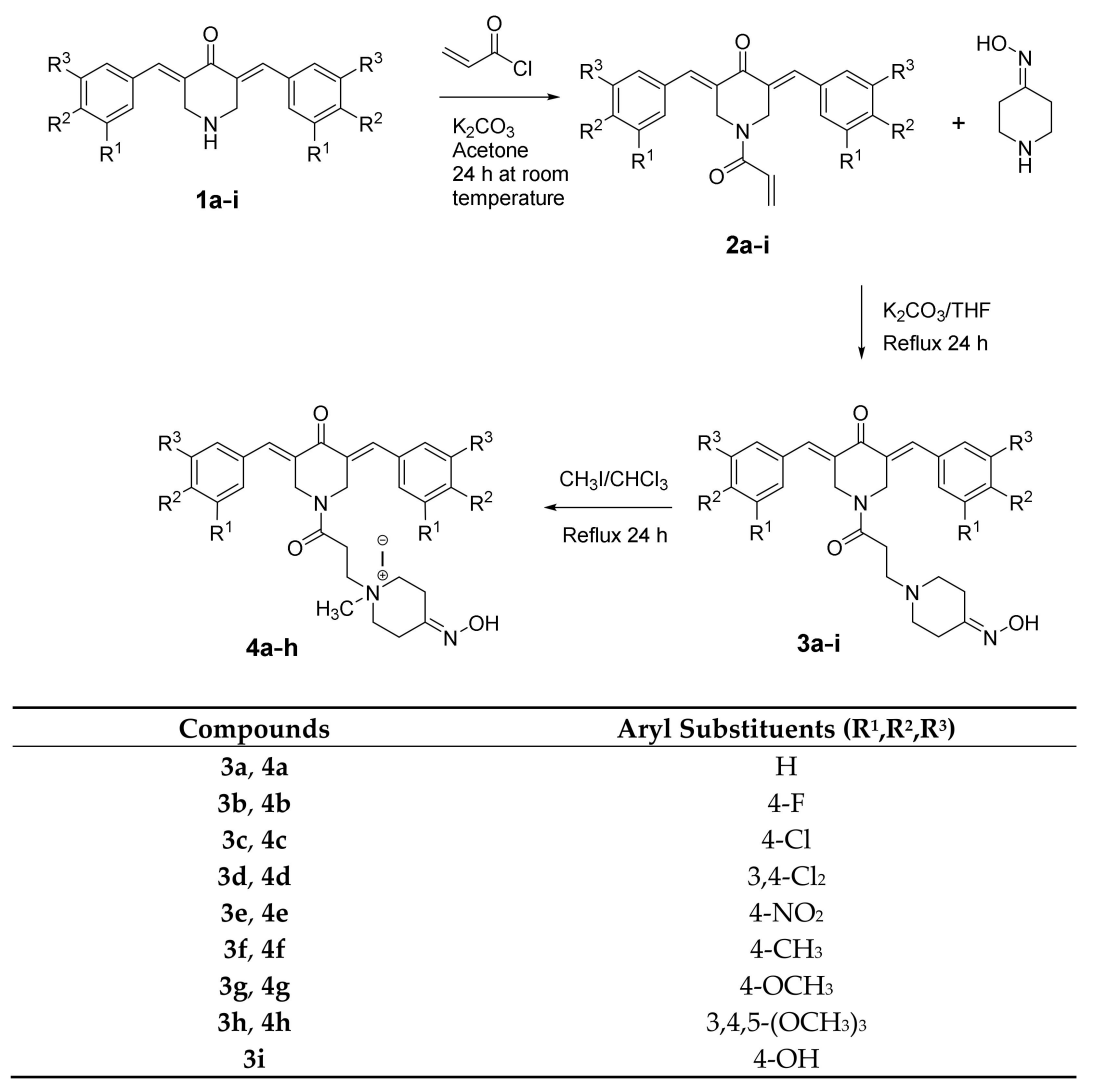
Synthetic chemical route for the compounds in series **3** and **4**.

**Figure 2:**
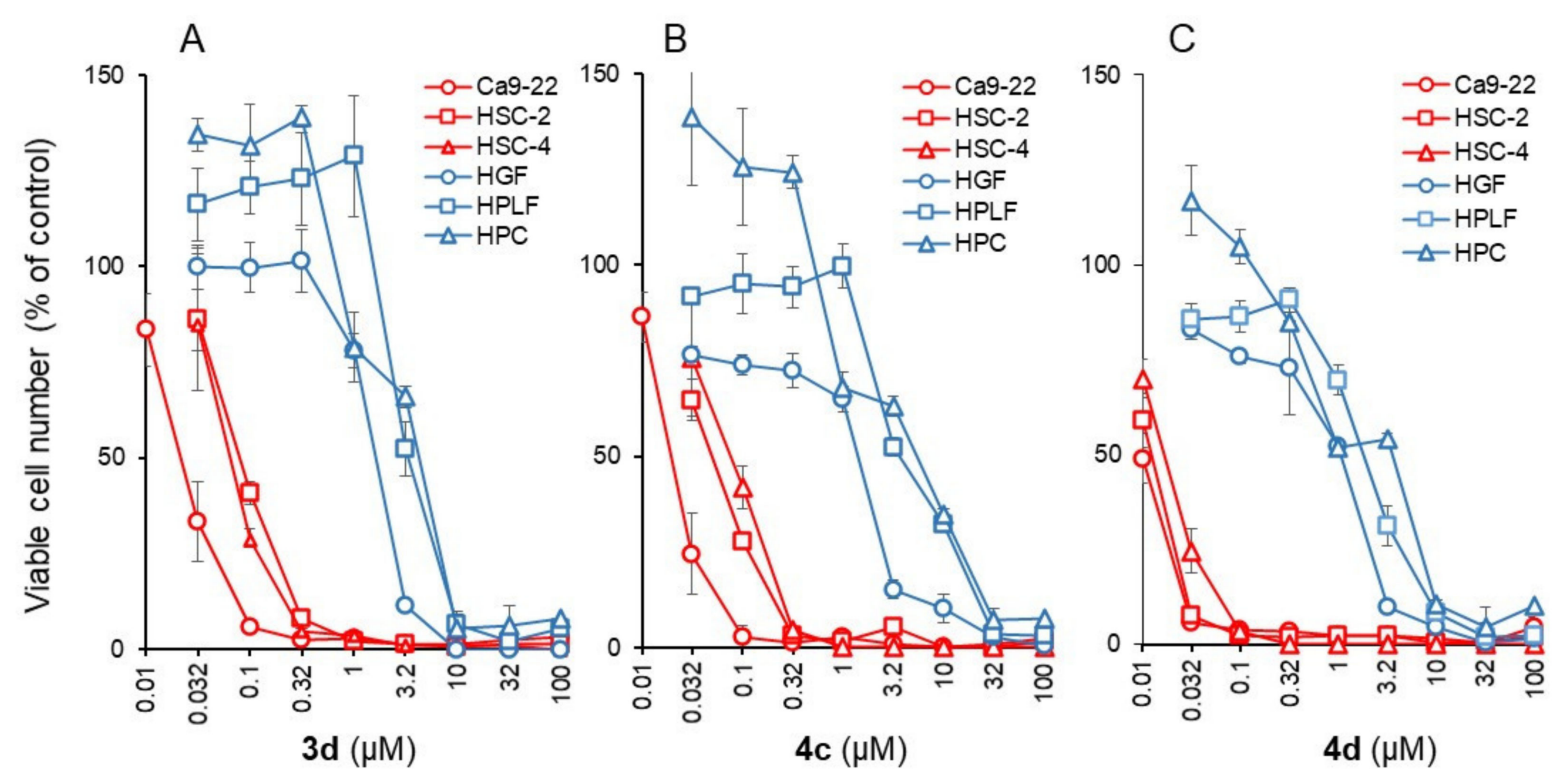
Dose–response curves of the cytotoxicity of three representative compounds. Human oral squamous cell carcinoma cell lines (Ca9-22, HSC-2, HSC-4) and human normal oral cells (HGF, HPLF, HPC) were incubated for 48 h without (control) or with the indicated concentrations of **3d** (**A**), **4c** (**B**) and **4d** (**C**), and the relative viable cell number was determined. Each value represents the mean ± S.D. of triplicate assays.

**Figure 3:**
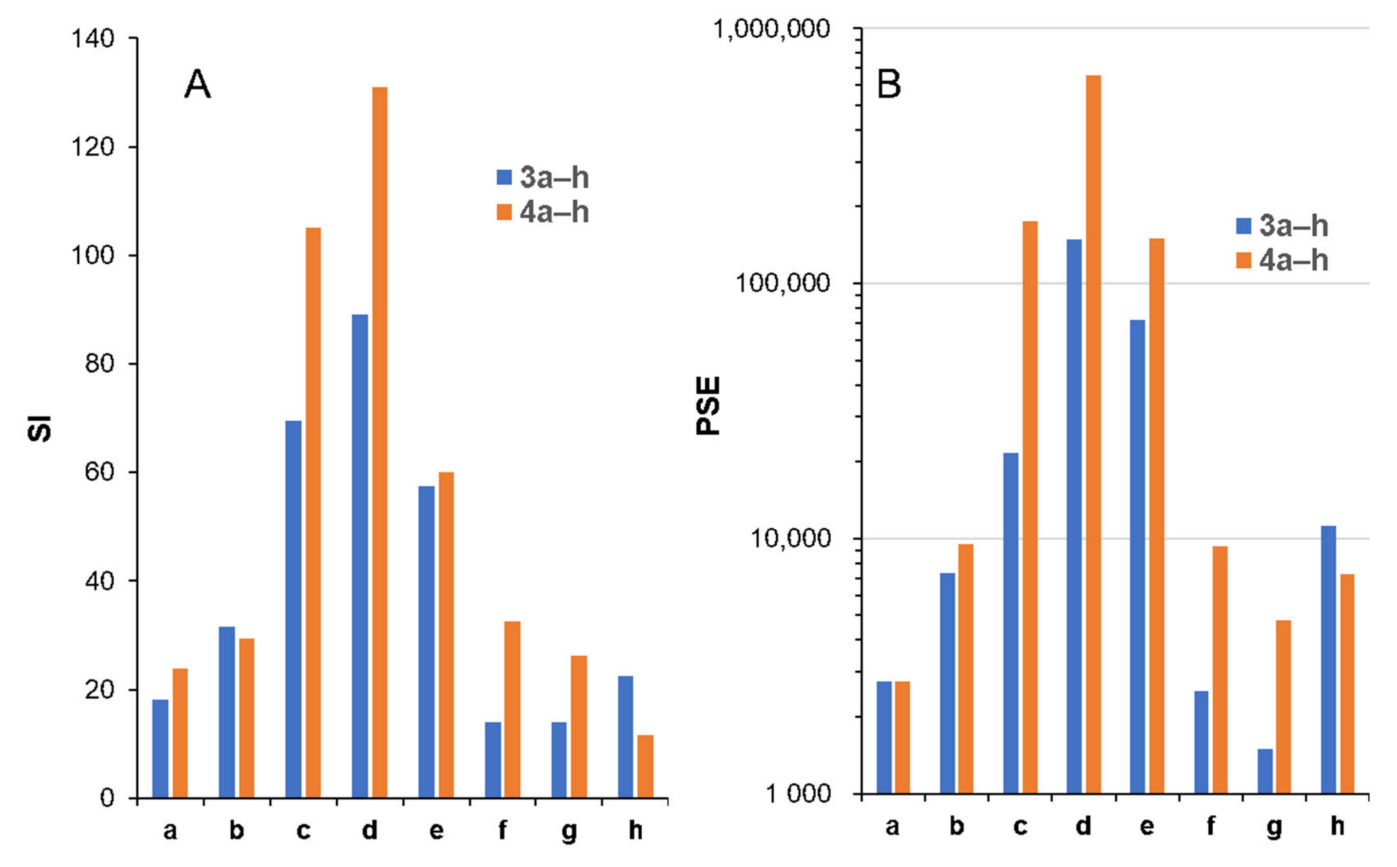
Comparison of the antitumor activity expressed as SI (**A**) and PSE (**B**) between series **3** (**3a**–**h**) and **4** (**4a**–**h**). These data are derived from Table 1 and Table 2.

**Figure 4:**
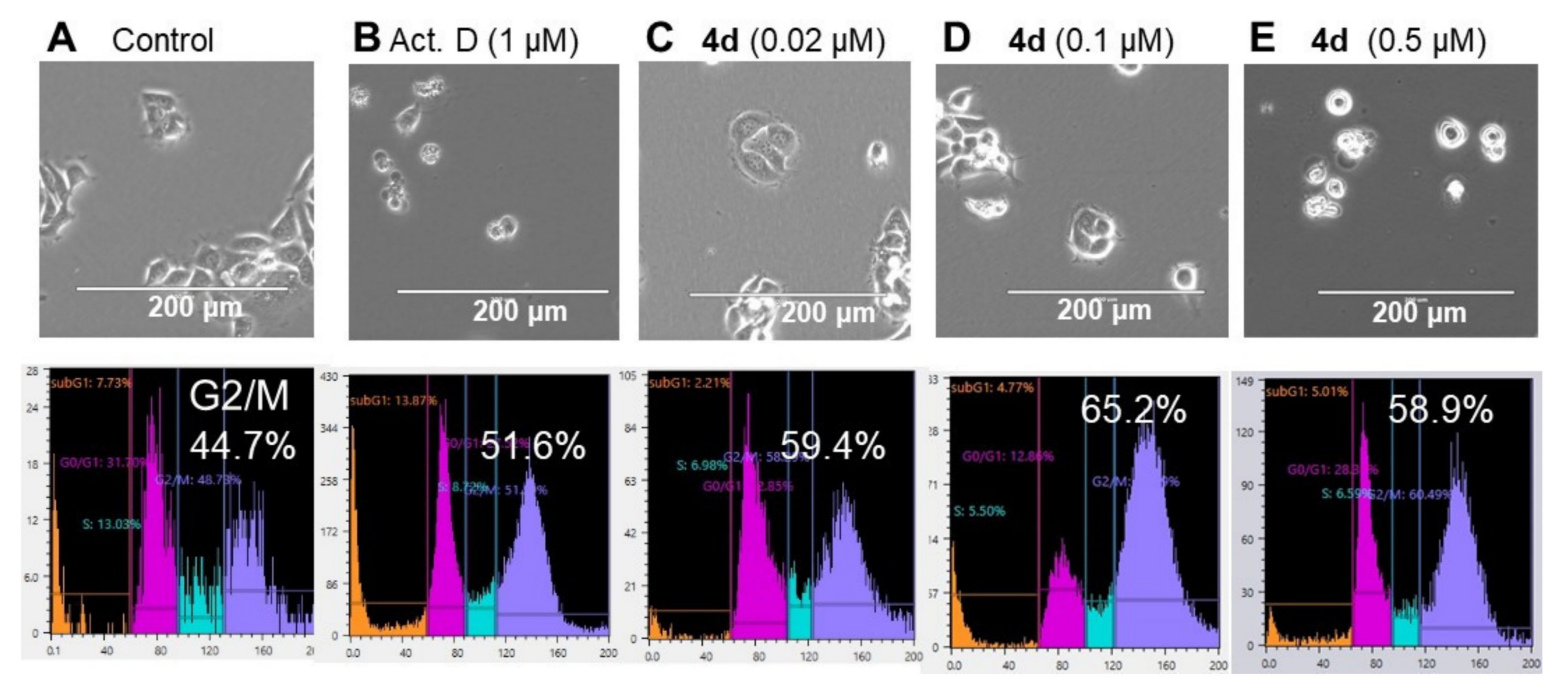
**4d** induced cell spreading and transient accumulation of the G2/M phase cells. Ca9-22 cells were treated for 24 h without (**A**), or with actinomycin D (1 μM) (**B**), **4d** (0.02, 0.1 or 0.5 μM) (**C**–**E**). (Upper column) Morphological changes. (Lower column) Cell cycle analysis.

**Figure 5:**
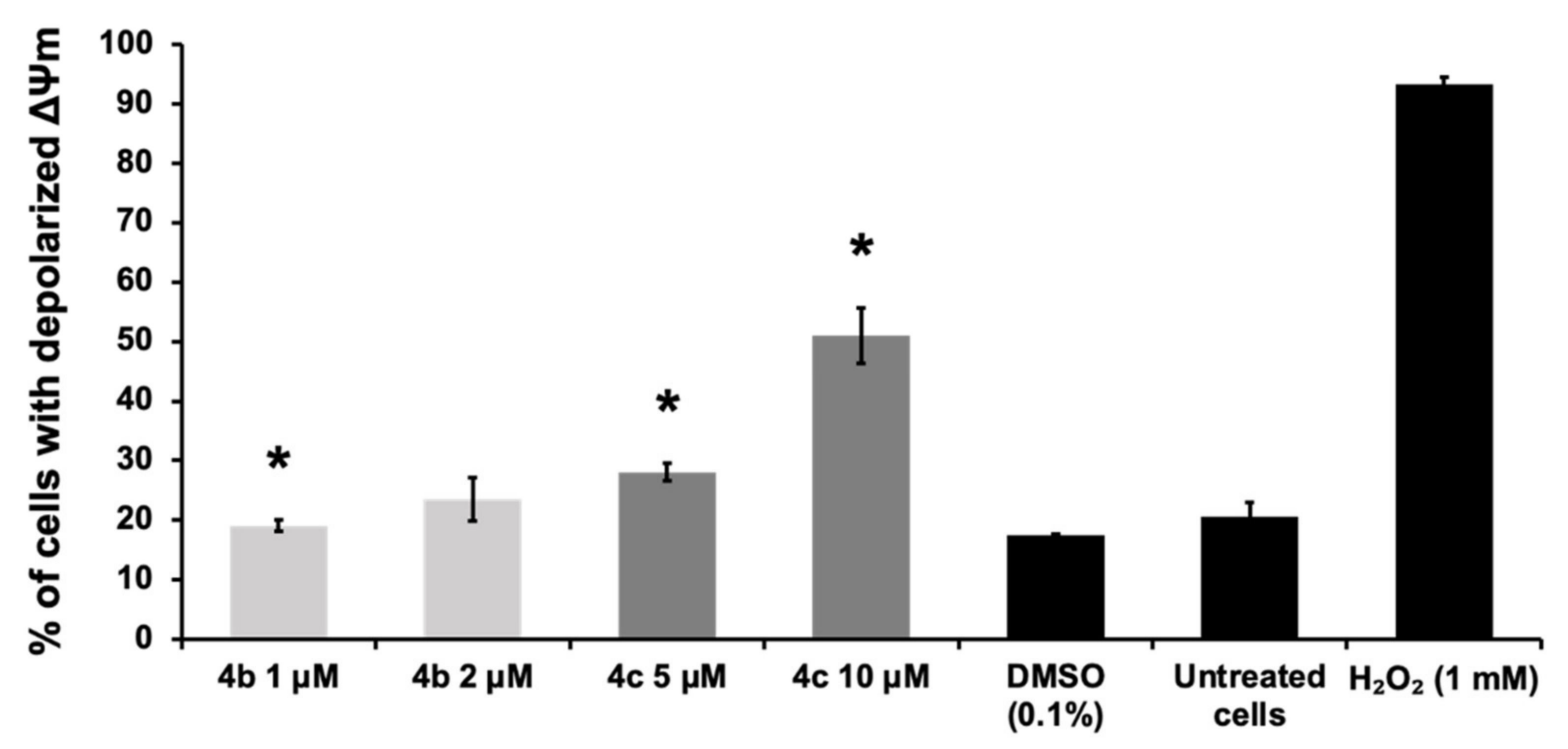
Effect of **4b** and **4c** on the mitochondrial membrane potential of CEM cells. The concentrations of **4b** and **4c** are the CC_50_ or twice the CC_50_ values. The asterisks above the bar graphs indicate that the *p*-value is less than 0.05 compared to dimethylsulfoxide.

The oximes **3a**–**i** and related quaternary ammonium salts **4a**–**h** were prepared by the methodology outlined in Scheme 1. These compounds were evaluated against human Ca9-22, HSC-2 and HSC-4 squamous cell carcinomas as well as human non-malignant gingival fibroblasts (HGF), human periodontal ligament fibroblasts (HPLF) and human pulp cells (HPC). These results are presented in Table 1 and Table 2. The dose–response curves of three representative compounds against these malignant and non-malignant cells are shown in Figure 2. A comparison of antitumor activity between **3a**–**h** and **4a**–**h** is presented in Figure 3. Cell cycle analysis and morphological changes induced in Ca9-22 cells by **4d**, the most active compound, are shown in Figure 4. In addition, the compounds in series **3** and **4** were evaluated against human CEM T-lymphoblastic leukemia cells and these data are presented in Table 3. Compounds **3a**–**h** were examined in the NCI in vitro screen and their efficacy towards leukemic and colon cancer cells is presented in Table 4 and Table 5, respectively. The lead compounds **3c**,**d** and **4c**–**e** were examined for drug-like properties and these results are presented in Table 6. Both **4b** and **4c** were examined for their effect on the MMP in CEM cells and the results are portrayed in Figure 5.

## 3. Discussion

The first stage of the synthesis of the compounds in series **3** and **4** was the acid-catalyzed condensation between a variety of aryl aldehydes and 4-piperidone, which led to **1a**–**I** [19]. *N*-Acylation of the enones in series **1** with acryloyl chloride generated **2a**–**i**. which reacted with 4-piperidone oxime to yield the desired products **3a**–**i**. Quaternization of **3a**–**h** with methyl iodide led to the formation of **4a**–**h**. Confirmation that methylation occurred on the piperidyl nitrogen atom was obtained using the heteronuclear multiple bond correlation (HMBC) technique. In this case, the HMBC cross peak of the exocyclic methylene group attached to the piperidyl nitrogen atom with the carbon atom of the methyl group was observed.

The cytotoxic properties of the compounds in series **3** and **4** towards neoplastic Ca9-22, HSC-2 and HSC-4 cells will be considered initially. These data are presented in Table 1. With the exception of **3i**, these compounds are potent cytotoxins. In fact, 90 % of the CC_50_ values of **3a**–**i** and **4a**–**h** are submicromolar and if one eliminates the outlier **3i**, the figure rises to 96%. Furthermore, 29% of the figures of **3a**–**i** and **4a**–**h** are in the double-digit nanomolar range (10^−8^ M). The average CC_50_ values of the enones in series **3** and **4** towards Ca9-22, HSC-2 and HSC-4 cells are presented in Table 1. The most potent compounds (average CC_50_ figures in µM in parentheses) are **3d** (0.06), **3e** (0.08), **4c** (0.06), **4d** (0.02) and **4e** (0.04), which are clearly lead molecules. These compounds were cytotoxic, rather than cytostatic, killing the cancer cells completely (Figure 2). The average CC_50_ figures of **3a**–**h** towards the three malignant cell lines is 0.40 µM and for **4a**–**h** the value is 0.29 µM suggesting that, in general, the quaternary ammonium compounds **4** are more cytotoxic than the precursor oximes **3**.

A comparison was made between the potencies of the compounds in series **3** and **4** with two established anticancer drugs melphalan and doxorubicin. Melphalan was chosen as a reference drug since it is an alkylating agent and the compounds in series **3** and **4** contain the 1,5-diaryl-3-oxo-1,4-pentadienyl group, which is considered to alkylate cellular thiols [20]. A number of antibiotics, such as doxorubicin, are potent anticancer drugs and compounds, which rival the efficacy of these two drugs and are useful lead molecules. The enones **3a**–**h** and **4a**–**h** are all substantially more potent than melphalan. For example, the average CC_50_ values of **3d** and **4d** are 445 and 1335 times lower than the corresponding figure for melphalan. Doxorubicin is much more potent than melphalan towards these cell lines with an average CC_50_ value of 0.24 µM. One may conclude that a number of compounds in series **3** and **4** exceed the potencies of these two anticancer agents towards the Ca9-22, HSC-2 and HSC-4 cell lines and are prototypes for analog development.

Comparisons were made between the potencies of the oximes **3a**–**h** and the related quaternary ammonium compounds **4a**–**h** when the same substituents are present in the aryl rings. Thus, the CC_50_ values of **3a** and **4a** in the Ca9-22 screen were compared and so forth. In these evaluations, standard deviations were taken into account. The following compounds have greater potencies (bioassay in parentheses), namely **4a**,**c**,**e**–**g** (Ca9-22), **4b**–**d**,**f**,**g** and **3a** (HSC-2) and **4c**,**d** (HSC-4). For the other comparisons, equipotency was observed. Hence, in 50% of the comparisons, the quaternary ammonium compounds in series **4** are more potent than the analogs in series **3**, while in 4% of the comparisons, an oxime has a lower CC_50_ value. Equipotency was noted in 46% of the comparisons made. This evaluation reveals that, in general, the quaternary ammonium salts are either more potent than the analogs in series **3** or are equipotent.

The stabilities of a representative oxime **3a** and the related quaternary ammonium compound **4a** were determined in a mixture of 9:1 deuterated dimethylsulfoxide and deuterium oxide. ^1^H NMR spectra were determined in dissolution and after 48 h incubation at 37 °C (the time and temperature of the cytotoxicity assays) and were found to be identical. Hence, cytotoxicity may be due to the compounds *per se* and not to any breakdown products.

A major issue in identifying novel lead molecules is whether tumour-selective toxicity is demonstrated, i.e., whether the compounds have greater toxicity to neoplasms than non-malignant cells. To this end, therefore, the enones in series **3** and **4** were evaluated against non-malignant HGF, HPLF and HPC cells and the data generated are presented in Table 2. Low toxicity is favoured and 29% of the CC_50_ values were above 10 µM. Once again, **3i** is an outlier displaying low cytotoxicity, which is conceivably due to the polar hydroxyl groups hindering penetration of the molecule into the cells. In terms of the other compounds, the lowest toxicity is demonstrated by **3a**–**c**,**g** and **4a**,**g** which have average CC_50_ values of greater than 10. These results compare favourably with the data for doxorubicin but not melphalan.

A further comparison was made between **3a**–**h** and **4a**–**h** in terms of their toxicity to HGF, HPLF and HPC non-malignant cells. In this case, the compounds with the higher CC_50_ values are preferable since they demonstrate less toxicity to normal cells. Comparisons were made between the CC_50_ values in each bioassay of the compounds in series **3** with the analog in series **4**, which has the same aryl substituents. The following compounds have higher CC_50_ values (bioassay in parentheses), namely **3b**–**d**, **3h**, **4a** (HGF), **3c**–**e**, **3h**,**4a** (HPLF) and **3d**,**e**,**4a** (HPC). In the remaining comparisons, equipotency was noted. Thus, in series **3** and **4**, higher CC_50_ values to HGF, HPLF and HPC cells were noted in 42% and 12% of the cases, respectively, while equal potency was noted in 46% of the comparisons. Hence, in general, the quaternary ammonium salts are more cytotoxic to non-malignant cells than the corresponding oximes. In summary, the quaternary ammonium salts are in general more toxic than the corresponding oximes to both Ca9-22, HSC-2 and HSC-4 neoplasms as well as HGF, HPLF and HPC non-malignant cells.

In order to assess whether the compounds in series **3** and **4** are more toxic to neoplasms than non-malignant cells, selective index (SI) figures were calculated. In the in vivo situation, a tumour is surrounded by a number of non-malignant cells. Hence in evaluating if compounds have greater toxicity for the tumours than the normal cells, SI values were obtained by dividing the average CC_50_ values for the HGF, HPLF and HPC cells by the CC_50_ figure against a specific neoplastic cell line. The data are presented in Table 1.

The SI values are all in excess of 1 indicating that the compounds in series **3** and **4** display greater toxicity towards the neoplasms than non-malignant cells. In particular, **3c**–**e** and **4c**–**e** have SI figures that are greater than 100 towards Ca9-22 cells. The average SI values are presented in Table 1, and **3c**–**e** and **4c**–**e** have figures in excess of 50. In particular, the average SI figures of **4c**,**d** are over 100, reemphasizing the identity of these two compounds as lead molecules.

A comparison was made between the SI data of the oximes **3a**–**h** and the quaternary ammonium salts **4a**–**h** when the aryl substituents are identical. Higher SI values were obtained in several cases (bioassay in parentheses), namely **4a**–**d**,**f**,**g** (Ca9-22), **4b**–**g** (HSC-2) and **4a**,**c**–**g** (HSC-4). Thus, in 71% of the comparisons made, greater selectivity was displayed by the compounds in series **4**.

In order to identify the most promising lead compounds in terms of both cytotoxic potencies and favourable SI values, potency–selectivity expression (PSE) values were computed. These data are the products of the reciprocal of the average CC_50_ value against Ca9-22, HSC-2 and HSC-4 cells and the average SI figure multiplied by 100. These values are presented in Table 2. Outstanding results may be noted for four compounds that have PSE values in excess of 100,000 namely **3d**, **4c**–**e**. A comparison between the PSE figures of **3a**–**h** and **4a**–**h** with the same substituents in the aryl rings revealed that higher figures were noted for **4a**–**g** than **3a**–**g** while **3h** has a higher PSE value than **4h**. This observation reinforces superior properties of the analogs in series **4** compared to the oximes **3** in general. However, the relative SI/PSE distribution patterns between series **3** and **4** (from **a** to **h**) are nearly superimposable with each other (Figure 3). The most active compound **4d** induced cell spreading due to G2/M cell accumulation without the induction of a subG1 population (Figure 4). It has been reported that 3,5-bis(3-iodo-5-methoxy-4-propoxybenzylidene)-*N*-acetylpiperidin-4-one inhibited tubulin and destabilization of microtubules, while 3,5-bis(3,4,5-trimethoxybenzylidene)-*N*-benzoylpiperidin-4-one destabilizes the microtubules [21]. Thus, it remained to investigate whether compound **4d** acts as an inhibitor of the microtubule. One may also note the very low PSE figure for **3i** revealing the negative influence on both cytotoxic potency and selectivity of a 4-hydroxy substituent in the aryl rings.

In view of the pronounced cytotoxic potencies of a number of the oximes and quaternary ammonium compounds described in this study, coupled with their high selectivity index figures, some guidelines were sought for the expansion of the series. First, an investigation was conducted to evaluate whether the magnitude of the electronic, hydrophobic, and steric properties of the aryl substituents correlated with cytotoxic potencies. Hence, linear and semi-logarithmic plots were made between the Hammett sigma (σ), Hansch pi (π) and molar refractivity (MR) constants and the CC_50_ values towards Ca9-22, HSC-2 and HSC-4 neoplastic cell lines.

The correlations (*p* < 0.05) and trends to correlations (*p* < 0.1) noted are presented in Table S1 in the Supplementary Section of this report. Negative correlations were noted between the magnitude of the σ values and the CC_50_ data generated by **3a**–**i**, **3a**–**h** (**3i** was considered an outlier) and **4a**–**h**. Thus, in the future, groups with strongl electron-attracting properties should be placed in the aryl rings. Apart from a trend towards a negative correlation between the CC_50_ values of **3a**–**i** towards HSC-4 cells and the π values of the aryl groups, no other correlations or trends to correlations were observed.

An important objective of the current investigation is to seek correlations between the SI values and the biodata generated. Consequently, linear and semilogarithmic plots were made between the σ, π and MR values of the aryl substituents and the SI values. These results are summarised in Table S2 in the Supplemental Section. The following positive correlations were noted between the σ values and the SI figures of **3a**–**i** in the Ca9-22, HSC-2 and HSC-4 screens, **3a**–**h** in the Ca9-22 and HSC-2 bioassays and **4a**–**h** towards HSC-2 and HSC-4 cells. In addition, positive correlations were found between the π constants and the SI data for **3a**–**i** towards Ca9-22 and **4a**–**h** in the Ca9-22, HSC-2 and HSC-4 screens. One may conclude that, in general, SI values rise as the magnitude of the σ values increases, and to a smaller extent, as the size of the π figures increase.

Thus, from considerations of the sizes of the σ, π and MR constants of the aryl rings, one may conclude that cytotoxic potencies and SI values are influenced principally by the σ constants and to a lesser degree by the π values. Hence in the future, compounds with strongly electron-attracting groups that are hydrophobic should be inserted into the aryl rings such as the 4-trifluoromethyl group (σ = 0.54, π = 0.88).[22].

The next phase of the investigation was to determine if the compounds in series **3** and **4** are cytotoxic to additional neoplastic cell lines. An evaluation was undertaken of the toxicity of **3** and **4** towards human CEM leukemic cells and the results are portrayed in Table 3. The data reveal that 77% of the IC_50_ values are below 10 µM, and the most potent analogs are the quaternary ammonium compounds **4b** and **4e**. Comparisons were made between the CC_50_ values of **3a**–**h** and **4a**–**h** when the same groups are present in the aryl rings. The data in Table 3 indicate that greater potencies were displayed by **3a**,**g,h** (38%) and **4b**–**f** (62%). Furthermore, the average CC_50_ values of **3a**–**h** and **4a**–**h** are 7.28 µM and 5.25 µM, respectively, which reveals that, in general, the compounds in series **4** are somewhat more potent than the related oximes in series **3**.

In view of the encouraging result with CEM leukemic cells, consideration was given to an assessment in the NCI in vitro screening program [23]. The oximes, but not the quaternary ammonium salts, were accepted for bioevaluation. Examination of the mean graphs [24] confirms the sensitivity of leukemic cells to these compounds. These data are presented in Table 4.

The results in Table 4 reveal that **3a**–**h** are potent inhibitors of the growth of a number of leukemic cell lines. No less than 78% of the IC_50_ values are submicromolar; in particular, **3c** has an average IC_50_ figure of 40 nM against SR leukemic cells. The compounds with the lowest average IC_50_ values are **3c** and **3h,** which have 4-chloro and 3,4,5-trimethoxy aryl substituents, respectively. A previous report indicated that melphalan, which is used in treating leukemias, has an average IC_50_ value against HL-60 (TB), K-562, RPMI-8226 and SR cells of 56.7 µM [25]. The compounds in series **3** are far more potent than melphalan.

The mean graphs generated in the NCI screen revealed that not only leukemic cells are very sensitive to **3a**–**h**, but these compounds are, in general, highly toxic towards colon cancers. These data are presented in Table 5. Some 70% of the IC_50_ values and 75% of the average IC_50_ values are submicromolar. The compounds with the lowest average IC_50_ values are **3c** and **3d**, namely compounds containing 4-chloro and 3,4-dichloro substituents, respectively. One particular reference drug is 5-fluorouracil, which is used in treating colon cancers. It has an average IC_50_ value of 8.46 µM against COLO 205, HCC2998, HCT-15, KM12 and SW-620 cells [25], which is substantially higher than the IC_50_ figures of the compounds in series **3**. Thus, the bioevaluation indicates the compounds in series **3** are novel potent cytotoxins.

A further question to resolve is whether the quaternary ammonium compounds have an effect on the mitochondrial membrane potential (MMP). Two compounds were chosen, namely **4b** and **4c**, which have a fivefold difference in potencies towards CEM cells as indicated in Table 3. Using CC_50_ and twice CC_50_ concentrations of **4b** and **4c** towards CEM cells revealed that both compounds interfered with the MMP (Figure 5). This effect is more noticeable with **4c**, suggesting this biochemical effect is a greater contributor to its cytotoxicity than is the case with **4b**. The isosteric replacement of the fluoro atom in **4b** by a chloro group to produce **4c** leads to differences not only in potency but also in the effect on the MMP. These observations illustrate the varying sensitivity of cells to different aryl substituents.

The biodata generated is encouraging. and in considering the future development of these compounds, an issue to be resolved is whether these compounds (and especially lead molecules) have drug-like properties. In terms of the potency, selectivity and PSE figures of the enones in series **3** and **4**, the most favourable biodata are displayed by **3c**,**d**,**4c**–**e**. In the case of good absorption, lead molecules are recommended to have a molecular weight not exceeding 500 and a logP value below 5 while the number of hydrogen bond acceptor and donor atoms should not exceed 10 and 5, respectively [26]. For good oral bioavailability, the number of rotatable bonds should be less than 10 and the polar surface area should not exceed 140 Å^2^ [27]. The relevant data for these compounds are presented in Table 6. The physicochemical data presented in Table 6 were generated using Swiss ADME [28]. In general, the lead compounds **3c**,**d**, **4c**–**e** show favorable properties except for their molecular weights. The oral bioavailability score of a compound is a prediction of its bioavailability in rats or permeability in Caco-2 cells. The compounds **3c**, **4c** and **4d** show favorable bioavailability scores. These assessments illustrate the need to proceed further with the development of this group of compounds.

## 4. Conclusions

A number of novel quaternary ammonium compounds **4a**–**h** and related oximes **3a**–**h** have been prepared. The perceived positive features of these compounds include the following characteristics. (1) The bioassays conducted reveal that these compounds are potent cytotoxins, which in general have much greater potencies than melphalan while some of these molecules exceed doxorubicin in potency. (2) The hypothesis that the quaternary ammonium salts **4a**–**h** would be more toxic to neoplasms than the related oximes **3a**–**h** is found to be true in the majority of cases. (3) The compounds in series **3** and **4** are more toxic to several neoplastic cell lines than to some non-malignant cells. (4) The molecules are structurally divergent from contemporary anticancer drugs and thus drug-resistant tumours may be sensitive to these compounds. (5) Several lead molecules, namely **3c**,**d**,**4c**–**e,** have been identified, which may serve as prototypes in further studies. (6) The lead molecules **3c**,**d**,**4c**–**e** have drug-like characteristics. (7) The design of the quaternary ammonium salts in series **4** included the possibility of the MMP as a cellular target at an early stage (8 h treatment) for these candidate antineoplastics. Both **4b** and **4c** caused a reduction in the MMP, which likely contributes to the cytotoxic effect. At a later stage (24 h), transient accumulation of cells at G2/M phase cells, without the induction of the subG1 population, was observed in Ca9-22 cells.

Expansion of the project can occur in several directions. In order to identify the lead compounds in series **3** and **4**, Table 7 was constructed. The substituents present in the most potent compounds in series **3** are **3c**–**e**,**h,** which have 4-chloro, 3,4-dichloro, 4-nitro and 3,4,5-trimethoxy substituents, respectively. In the case of the quaternary ammonium compounds, **4b**,**d**, **e** are optimal, i.e., the 4-fluoro, 3,4-dichloro and 4-nitro groups, respectively. Hence in the future, a single bromo group, multiple halogens (fluoro and chloro substituents) and other electron-attracting substituents such as the trifluoromethyl group should be placed in the aryl rings. Clearly, electron-donating groups should be avoided; this assessment is borne by the introduction of the 4-dimethylamino group into the aryl rings of **3i**.

Potency may be increased further still with the acylation of the oxime hydroxyl group with acryloyl and chloroacetyl groups to create compounds with increased thiol alkylating properties. Prodrug formations should be considered, such as forming thiol adducts at the olefinic carbon atoms, which may slowly release the pharmacophoric group. When further amplification has been undertaken and the biodata are to hand, one or more lead compounds will undergo further scrutiny, which will address such issues as the solubility, pharmacokinetic and stability properties of the molecules. A number of quaternary ammonium compounds target the lysosomes [29] and hence future studies with various compounds in series **4** or modifications thereof should be assayed to determine whether they target lysosomes.

Comparisons were made between the cytotoxic potencies of the compounds in series **3** and **4** with studies undertaken on related compounds. Only general observations can be made since different neoplastic cell lines were employed in the cytotoxic assays. In general, the absence of an *N*-acyl group of the 3,5-bis(benzylidene)-4-piperidones gave rise to compounds that are less potent cytotoxic agents than the compounds in series **3** and **4** [19,30]. The placement of a methyl group on the piperidyl nitrogen atom also led to compounds with lower cytotoxic potencies than **3a**–**h** and **4a**–**h** [19,31]. On the other hand, *N*-acylation, in general, gave rise to a series of compounds that have similar activity as was found in series **3** and **4** [32].

## 5. Experimental Methods

### 5.1. Syntheses of 3a–i, 4a–h

The ^1^H and ^13^C NMR spectra were determined using a Bruker Avance AMX 500 FT instrument (Billerica, MA, USA) equipped with a BBO probe. Chemical shifts (δ) are reported in ppm. Mass spectra were obtained using a JEOL JMS-7100 GCV Accu tof-gc V4G instrument (Peabody, MA, USA). Melting points were determined using a DigiMelt-MPA160 instrument (Sunnyvale, CA, USA).

4-Piperidone oxime was prepared as follows. A solution of sodium hydroxide (7.81 g, 195.3 mmol) in water (100 mL) was added to a mixture of hydroxylamine hydrochloride (6.79 g, 97.65 mmol) in ethanol (200 mL) and stirred at room temperature for 0.25 h. Then 4-piperidone hydrochloride monohydrate (10 g, 65.1 mmol) was added and the mixture was heated under reflux for 2 h. The solvent was removed, and water (250 mL) was added to the residue. The desired compound was extracted with ethyl acetate (250 mL × 3). The organic extracts were washed with water and brine and dried over anhydrous sodium sulfate. Removal of the solvent gave 4-piperidone oxime [33].

Yield 52%. MP: 236 °C (dec.). ^1^H NMR (500 MHz, DMSO-*d*_6_) δ ppm 1.73 (s, 1 H) 2.07–2.11 (m, 2 H) 2.33–2.38 (m, 2 H) 2.68 (t, *J* = 5.9 Hz, 2 H) 2.75 (t, *J* = 5.8 Hz, 2 H).

#### 5.1.1. Synthesis of Compounds **2a**–**i**

The 3,5-bis(benzylidene)-4-piperidones **1a–i** were prepared by a method found in the literature [19]. Aryl aldehyde (11.46 mmol) and 4-piperidone hydrochloride monohydrate (5.60 mmol) were added to glacial acetic acid (15 mL) and stirred for five minutes. Dry hydrogen chloride gas was passed through this mixture for about 45 min until a clear solution was obtained. The reaction mixture was stirred at room temperature for 24 h. The precipitate formed in the reaction was collected by filtration and was treated with a mixture of 10 mL of saturated aqueous potassium carbonate solution (25% *w*/*v*) and 10 mL of acetone and stirred at room temperature for 45 min. The free base was collected by filtration, washed with ice-cold water and dried under vacuum to afford **1a**–**i** as yellow solids [19]. The structures were confirmed by ^1^H NMR spectroscopy.

Acryloyl chloride (0.9 mL, 10.9 mmol) in acetone (1 mL) was added dropwise to a stirring mixture of 3,5-diarylidenepiperidin-4-ones (2.0 g, 7.26 mmol), potassium carbonate (1.61 g, 11.62 mmol) and acetone (15 mL) in an ice bath. The reaction continued for 24 h at ambient temperature. After the starting material was completely consumed, the reaction mixture was poured into ice. The precipitate obtained was filtered and washed with water to afford the appropriate 1-acryloyl-3,5-bis(benzylidene)-4-piperidones **2a–i** as yellow solids. These compounds were identified using ^1^H NMR spectroscopy.

#### 5.1.2. Synthesis of Compounds **3a–i**

To a mixture of 4-piperidone-oxime (0.41 g, 3.64 mmol) and dry potassium carbonate (0.84 g, 6.07 mmol) in anhydrous tetrahydrofuran (10 mL), the appropriate 1-acryloyl-3,5-bis(benzylidene)-4-piperidone **2** (1.0 g, 3.04 mmol) was added and stirred at reflux temperature for 24 h. After the disappearance of the starting material, the solvent was completely evaporated, and the residue obtained was washed with cold water. Alternatively, the compounds **3a**–**i** can also be synthesized in the absence of a base by mixing the two starting materials in ethanol, stirred overnight at reflux temperature, and evaporating the ethanol to get the products. The obtained products **3a**–**i** were vacuum dried and recrystallized in ethanol. The compounds **3a**–**d**,**f**,**g** were dissolved in a mixture of chloroform (10 mL) and ethanol (10 mL) and converted into hydrochlorides by passing dry hydrogen chloride gas into the solution for 1h and evaporating the solvent under vacuum. The corresponding hydrochlorides were recrystallized from ethanol.

**(1)**  **(3~{E},5~{E})-3,5-bis-benzylidine-1-[3-(4-hydroximino-1-piperidyl)propanoyl]piperidin-4-one hydrochloride (3a)**

Yield: 88%. MP: 172.3 °C. ^1^H NMR (500 MHz, DMSO-*d*_6_) δ ppm 2.00–2.07 (m, 2 H) 2.09–2.16 (m, 2 H) 2.16–2.22 (m, 2 H) 2.27–2.35 (m, 4 H) 2.36–2.41 (m, 2 H) 4.81–4.88 (m, 4 H) 7.46–7.55 (m, 6 H) 7.56–7.59 (m, 4 H) 7.68–7.74 (m, 2 H) 10.25 (s, 1 H). ^13^C NMR (125 MHz, DMSO-*d*_6_) δ ppm 23.98, 30.93, 42.70, 46.24, 51.58, 52.98, 128.88, 129.63, 130.56, 132.69, 132.89, 134.16, 134.38, 135.93, 136.40, 154.37, 170.16, 186.22. MS (FD) *m*/*z* found: 443.2231 (M+), 444.2283 (M + H), Calc: 443.2209.

**(2)**  **(3~{E},5~{E})-3,5-bis[(4-fluorophenyl)methylene]-1-[3-(4-hydroximino-1-piperidyl)propanoyl]piperidin-4-one hydrochloride (3b)**

Yield: 84%. MP: 213.4 °C. ^1^H NMR (500 MHz, DMSO-*d*_6_) δ ppm 2.03–2.07 (m, 2 H) 2.15 (t, *J* = 5.9 Hz, 2 H) 2.21 (t, *J* = 5.9 Hz, 2 H) 2.29–2.36 (m, 4 H) 2.37–2.42 (m, 2 H) 4.82 (s, 4 H) 7.36 (t, *J* = 8.8 Hz, 4 H) 7.65 (t, *J* = 6.6 Hz, 4 H) 7.70 (s, 2 H) 10.26 (br s, 1 H). ^13^C NMR (125 MHz, DMSO-*d*_6_) δ ppm 23.95, 25.56, 30.25, 30.92, 42.54, 46.15, 51.66, 53.00, 53.03, 114.54, 115.84, 116.01, 130.73, 130.94, 132.45, 132.67, 132.97, 133.04, 134.83, 135.23, 154.19, 161.58, 163.56, 163.65, 170.20, 174.56, 186.15. MS (FD) *m*/*z* found: 479.2060 (M+), 480.2090 (M + H), Calc: 479.2020.

**(3)**  **(3~{E},5~{E})-3,5-bis[(4-chlorophenyl)methylene]-1-[3-(4-hydroximino-1-piperidyl)propanoyl]piperidin-4-one hydrochloride (3c)**

Yield: 79%. MP: 167.9 °C. ^1^H NMR (500 MHz, DMSO-*d*_6_) δ ppm 2.04–2.07 (m, 2 H) 2.15 (t, *J* = 5.9 Hz, 2 H) 2.20 (t, *J* = 5.8 Hz, 2 H) 2.30–2.35 (m, 4 H) 2.37–2.42 (m, 2 H) 4.81 (s, 4 H) 7.57–7.63 (m, 8 H) 7.68 (s, 2 H) 10.25 (s, 1 H). ^13^C NMR (125 MHz, DMSO-*d*_6_) δ ppm 42.71, 55.09, 62.30, 113.53, 114.04, 127.13, 128.78, 132.54, 158.50, 168.78, 185.92. MS (FD) *m*/*z* found: 511.1455 (M+), 512.1507 (M + H), Calc: 511.1429.

**(4)**  **(3~{E},5~{E})-3,5-bis[(3,4-dichlorophenyl)methylene]-1-[3-(4-hydroximino-1-piperidyl)propanoyl]piperidin-4-one hydrochloride (3d)**

Yield 82%. MP: 194.1 °C. ^1^H NMR (500 MHz, DMSO-*d*_6_) δ ppm 2.05–2.09 (m, 2 H) 2.18 (t, *J* = 5.9 Hz, 2 H) 2.23 (t, *J* = 5.8 Hz, 2 H) 2.31–2.37 (m, 4 H) 2.38–2.43 (m, 2 H) 4.80 (s, 4 H) 7.57 (dd, *J* = 8.4, 1.9 Hz, 2 H) 7.66 (s, 2 H) 7.78 (d, *J* = 8.4 Hz, 2 H) 7.9 (s, 2 H) 10.25 (s, 1 H). ^13^C NMR (125 MHz, DMSO-*d*_6_) δ ppm 23.98, 30.25, 30.93, 39.03, 42.44, 46.06, 51.72, 53.00, 53.04, 130.28, 130.91, 131.60, 131.62, 131.67, 132.16, 132.24, 133.66, 133.95, 134.18, 134.36, 134.82, 134.98, 154.27, 170.32, 185.96. MS (FD) *m*/*z* found: 579.0657 (M+), 580.0621 (M + H), Calc: 579.0650.

**(5)**  **(3~{E},5~{E})-1-[3-(4-hydroximino-1-piperidyl)propanoyl]-3,5-bis[(4-nitrophenyl)methylene]piperidin-4-one (3e)**

Yield 72%. MP: 114.1 °C. ^1^H NMR (500 MHz, DMSO-*d*_6_) δ ppm 2.00–2.04 (m, 2 H) 2.12–2.17 (m, 2 H) 2.20 (t, *J* = 5.8 Hz, 2 H) 2.28 (t, *J* = 5.9 Hz, 2 H) 2.32–2.40 (m, 4 H) 4.87 (d, *J* = 8.2 Hz, 4 H) 7.80 (s, 2 H) 7.86 (br s, 4 H) 8.34 (d, *J* = 8.5 Hz, 4 H) 10.27 (s, 1 H). ^13^C NMR (125 MHz, DMSO-*d*_6_) δ ppm 18.59, 23.90, 30.20, 30.85, 42.50, 46.19, 51.73, 52.75, 52.93, 53.08, 53.20, 56.05, 109.58, 114.54, 123.79, 131.54, 131.68, 133.87, 134.15, 135.51, 135.63, 140.63, 140.78, 147.38, 154.26, 170.36, 186.11. MS (FD) *m*/*z* found: 533.1928 (M+), 534.1999 (M + H), Calc: 533.1910.

**(6)**  **(3~{E},5~{E})-3,5-bis[(4-methylphenyl)methylene]-1-[3-(4-hydroximino-1-piperidyl)propanoyl]piperidin-4-one hydrochloride (3f)**

Yield 91%. MP: 195.4 °C. ^1^H NMR (500 MHz, DMSO-*d*_6_) δ ppm 2.01–2.07 (m, 2 H) 2.10 (t, *J* = 5.9 Hz, 2 H) 2.18 (t, *J* = 5.7 Hz, 2 H) 2.27–2.34 (m, 4 H) 2.37 (br s, 2 H) 2.38 (s, 6 H) 4.82 (d, *J* = 8.7 Hz, 4 H) 7.34 (d, *J* = 8.0 Hz, 4 H) 7.47 (d, *J* = 8.0 Hz, 4 H) 7.68 (s, 2 H) 10.25 (s, 1 H). ^13^C NMR (125 MHz, DMSO-*d*_6_) δ ppm 21.05, 23.96, 30.23, 30.93, 39.03, 42.80, 46.24, 51.50, 52.95, 52.98, 113.86, 129.49, 130.61, 130.68, 131.41, 131.65, 131.90, 132.14, 135.84, 136.41, 139.62, 139.65, 154.33, 170.08, 186.04. MS (FD) *m*/*z* found: 471.2544 (M+), 472.2589 (M + H), Calc: 471.2528.

**(7)**  **(3~{E},5~{E})-3,5-bis[(4-methoxyphenyl)methylene]-1-[3-(4-hydroximino-1-piperidyl)propanoyl]piperidin-4-one hydrochloride (3g)**

Yield 74%. MP: 186.2 °C. ^1^H NMR (500 MHz, DMSO-*d*_6_) δ ppm 2.01–2.09 (m, 2 H) 2.14 (t, *J* = 5.9 Hz, 2 H) 2.20 (t, *J* = 5.8 Hz, 2 H) 2.26–2.32 (m, 2 H) 2.32–2.45 (m, 2 H) 3.84 (s, 6 H) 4.82 (d, *J* = 6.4 Hz, 4 H) 7.08 (d, *J* = 8.8 Hz, 4 H) 7.55 (d, *J* = 8.3 Hz, 4 H) 7.66 (s, 2 H) 10.24 (s, 1 H). ^13^C NMR (125 MHz, DMSO-*d*_6_) δ ppm 20.87, 23.97, 30.34, 30.93, 42.37, 42.77, 46.29, 47.01, 51.59, 53.00, 53.05, 55.37, 114.41, 126.75, 127.01, 130.42, 130.58, 130.87, 132.59, 132.62, 135.58, 135.71, 135.96, 136.10, 154.36, 160.37, 168.63, 170.08, 185.82. MS (FD) *m*/*z* found: 503.2430 (M+), 504.2478 (M + H), Calc: 503.2420.

**(8)**  **(3~{E},5~{E})-1-[3-(4-hydroximino-1-piperidyl)propanoyl]-3,5-bis[(3,4,5-trimethoxyphenyl)methylene]piperidin-4-one (3h)**

Yield 71%. MP: 177.8 °C. ^1^H NMR (500 MHz, DMSO-*d*_6_) δ ppm 2.05 (t, *J* = 5.9 Hz, 2 H) 2.18 (t, *J* = 5.9 Hz, 2 H) 2.23 (t, *J* = 5.7 Hz, 2 H) 2.30–2.33 (m, 2 H) 2.41 (s, 4 H) 3.74 (s, 6 H) 3.85 (d, *J* = 5.6 Hz, 12 H) 4.88 (s, 4 H) 6.89 (s, 2 H) 6.87 (s, 2 H) 7.68 (s, 2 H) 10.24 (s, 1 H). ^13^C NMR (125 MHz, DMSO-*d*_6_) δ ppm 23.96, 30.47, 30.90, 42.62, 46.37, 51.66, 53.00, 56.08, 60.17, 108.10, 108.15, 129.71, 129.93, 131.99, 132.22, 136.41, 136.75, 138.79, 152.89, 154.28, 170.31, 185.99. MS (FD) *m*/*z* found: 623.2846 (M+), 624.2875 (M + H), Calc: 623.2843.

**(9)**  **(3~{E},5~{E})-3,5-bis[(4-hydroxyphenyl)methylene]-1-[3-(4-hydroximino-1-piperidyl)propanoyl]piperidin-4-one (3i)**

Yield 68%. MP: 216.6 °C. ^1^H NMR (500 MHz, DMSO-*d*_6_) δ ppm 2.05 (t, *J* = 5.8 Hz, 2 H) 2.17 (t, *J* = 5.9 Hz, 2 H) 2.22 (t, *J* = 5.7 Hz, 2 H) 2.29–2.33 (m, 2 H) 2.34–2.42 (m, 4 H) 4.80 (d, *J* = 6.3 Hz, 4 H) 6.90 (d, *J* = 8.5 Hz, 4 H) 7.43 (dd, *J* = 8.0, 3.74 Hz, 4 H) 7.61 (s, 2 H) 10.13 (br s, 1 H) 10.27 (s, 1 H) ^13^C NMR (125 MHz, DMSO-*d*_6_) δ ppm 24.00, 30.39, 30.93, 42.84, 46.32, 55.20, 55.91, 75.00, 117.23, 118.91, 126.05, 126.28, 128.40, 128.43, 128.47, 128.50, 128.75, 128.80, 129.44, 129.52, 130.45, 130.55, 132.91, 133.33, 134.40, 134.49, 135.48, 136.07, 136.86, 137.15, 141.92, 145.09, 170.59, 186.39. MS (FD) *m*/*z* found: 604.2734 (M+), 605.2767 (M + H), Calc: 604.2726.

#### 5.1.3. Synthesis of Compounds **4a**–**h**

The appropriate compound in series **3** (1.13 mmol) was added to a solution of methyl iodide (0.24 g, 1.69 mmol) in chloroform (10 mL). The mixture was heated under reflux for 24 h after which time the solvent was evaporated and **4a**–**h** were recrystallized from ethanol.

**(1)**  **(3~{E},5~{E})-3,5-bis-benzylidine-1-[3-(4-hydroximino-1-piperidyl)propanoyl]piperidin-4-one methiodide (4a)**

Yield 81%. MP: 173 °C. ^1^H NMR (500 MHz, DMSO-*d*_6_) δ ppm 2.56–2.68 (m, 2 H) 2.86–2.92 (m, 2 H) 2.95 (t, *J* = 7.7 Hz, 2 H) 3.02 (s, 3 H) 3.36–3.49 (m, 4 H) 3.59 (t, *J* = 7.5 Hz, 2 H) 4.85 (br s, 2 H) 4.95 (br s, 2 H) 7.46–7.59 (m, 8 H) 7.64 (d, *J* = 5.8 Hz, 2 H) 7.66 (br s, 1 H) 7.72 (s, 1 H) 10.94 (s, 1 H). ^13^C NMR (125 MHz, DMSO-*d*_6_) δ ppm 18.69, 24.79, 25.22, 41.85, 46.16, 58.05, 59.12, 59.16, 128.80, 128.93, 129.54, 129.68, 130.33, 130.84, 132.40, 132.56, 134.16, 134.24, 135.83, 136.29, 148.05, 167.51, 186.71. MS (FD) *m*/*z* found: 458.2461 (M+), 459.2502 (M + H), Calc: 458.2444.

**(2)**  **(3~{E},5~{E})-3,5-bis[(4-fluorophenyl)methylene]-1-[3-(4-hydroximino-1-piperidyl)propanoyl]piperidin-4-one methiodide (4b)**

Yield 86%. MP: 163 °C. ^1^H NMR (500 MHz, DMSO-*d*_6_) δ ppm 2.57–2.70 (m, 2 H) 2.90 (d, *J* = 16.6 Hz, 2 H) 2.94–2.99 (m, 2 H) 3.04 (s, 3 H) 3.39–3.49 (m, 4 H) 3.60 (t, *J* = 7.2 Hz, 2 H) 4.81 (br s, 2 H) 4.92 (br s, 2 H) 7.38 (d, *J* = 8.5 Hz, 4 H) 7.64 (br s, 2 H) 7.72 (d, *J* = 8.8 Hz, 4 H) 10.95 (s, 1 H). ^13^C NMR (125 MHz, DMSO-*d*_6_) δ ppm 18.73, 24.83, 25.25, 41.66, 46.17, 58.10, 59.15, 59.20, 113.84, 115.83, 115.93, 116.01, 116.10, 130.82, 130.85, 132.24, 132.74, 132.81, 133.35, 133.42, 134.73, 135.30, 148.13, 161.63, 163.61, 167.60, 186.60. MS (FD) *m*/*z* found: 494.2238(M+), 495.2306(M + H), Calc: 494.2250.

**(3)**  **(3~{E},5~{E})-3,5-bis[(4-chlorophenyl)methylene]-1-[3-(4-hydroximino-1-piperidyl)propanoyl]piperidin-4-one methiodide (4c)**

Yield 89%. MP: 192 °C. ^1^H NMR (500 MHz, DMSO-*d*_6_) δ ppm 2.53–2.56 (m, 2 H) 2.57–2.69 (m, 2 H) 2.87–2.92 (m, 2 H) 2.95 (t, *J* = 7.7 Hz, 2 H) 3.04 (s, 3 H) 3.37–3.50 (m, 2 H) 3.59 (t, *J* = 7.6 Hz, 2 H) 4.81 (s, 2 H) 4.92 (br s, 2 H) 7.57–7.65 (m, 6 H) 7.68 (d, *J* = 8.8 Hz, 4 H) 10.95 (s, 1 H). ^13^C NMR (125 MHz, DMSO-*d*_6_) δ ppm 18.73, 24.83, 25.23, 41.70, 46.13, 58.10, 59.15, 59.20, 79.21, 114.16, 128.90, 128.97, 132.10, 132.64, 132.94, 133.08, 133.14, 134.30, 134.44, 134.60, 135.16, 148.14, 167.62, 186.51. MS (FD) *m*/*z* found: 526.1652 (M+), 527.1656 (M + H), Calc: 526.1659.

**(4)**  **(3~{E},5~{E})-3,5-bis[(3,4-dichlorophenyl)methylene]-1-[3-(4-hydroximino-1-piperidyl)propanoyl]piperidin-4-one methiodide (4d)**

Yield 88%. MP: 177.2 °C. ^1^H NMR (500 MHz, DMSO-*d*_6_) δ ppm 2.54 (d, *J* = 5.7 Hz, 2 H) 2.58–2.70 (m, 2 H) 2.88–2.93 (m, 2 H) 2.96 (t, *J* = 7.6 Hz, 2 H) 3.05 (s, 3 H) 3.37–3.50 (m, 2 H) 3.60 (t, *J* = 7.6 Hz, 2 H) 4.79 (br s, 2 H) 4.92 (br s, 2 H) 7.57 (d, *J* = 8.1 Hz, 1 H) 7.60–7.65 (m, 2 H) 7.67 (s, 1 H) 7.79 (dd, *J* = 8.0, 2.40 Hz, 2 H) 7.90 (s, 1 H) 7.94 (s, 1 H) 10.95 (s, 1 H). ^13^C NMR (125 MHz, DMSO-*d*_6_) δ ppm 18.74, 24.83, 25.26, 41.69, 46.19, 58.10, 59.11, 59.20, 115.70, 130.17, 130.42, 130.98, 131.59, 131.67, 131.93, 132.13, 132.23, 132.58, 133.46, 133.86, 134.06, 134.19, 134.86, 134.92, 148.12, 167.72, 186.29. MS (FD) *m*/*z* found: 594.0859 (M+), 595.0864 (M + H), Calc: 594.0879.

**(5)**  **(3~{E},5~{E})-1-[3-(4-hydroximino-1-piperidyl)propanoyl]-3,5-bis[(4-nitrophenyl)methylene]piperidin-4-one methiodide (4e)**

Yield 77%. MP: 189.8 °C. ^1^H NMR (500 MHz, DMSO-*d*_6_) δ ppm 2.56–2.68 (m, 2 H) 2.89 (dt, *J* = 16.8, 5.3 Hz, 2 H) 2.95 (t, *J* = 7.7 Hz, 2 H) 3.02 (s, 3 H) 3.38–3.50 (m, 4 H) 3.58 (t, *J* = 7.7 Hz, 2 H) 4.85 (br s, 2 H) 4.98 (br s, 2 H) 7.75 (br s, 1 H) 7.81 (br s, 1 H) 7.85 (d, *J* = 8.5 Hz, 2 H) 7.92 (d, *J* = 8.8 Hz, 2 H) 8.35 (d, *J* = 7.3 Hz, 4 H) 10.94 (s, 1 H). ^13^C NMR (125 MHz, DMSO-*d*_6_) δ ppm 18.72, 24.80, 25.24, 41.72, 46.19, 58.10, 59.03, 59.20, 114.06, 123.81, 131.39, 131.87, 133.78, 134.34, 135.09, 135.46, 140.61, 140.75, 147.42, 148.12, 167.75, 186.43. MS (FD) *m*/*z* found: 548.2114(M+), 549.2147 (M + H), Calc: 548.2145.

**(6)**  **(3~{E},5~{E})-3,5-bis[(4-methylphenyl)methylene]-1-[3-(4-hydroximino-1-piperidyl)propanoyl]piperidin-4-one methiodide (4f)**

Yield 95%. MP: 183.9 °C. ^1^H NMR (500 MHz, DMSO-*d*_6_) δ ppm 2.39 (d, *J* = 6.8 Hz, 2 H) 2.49 (s, 6 H) 2.56–2.67 (m, 2 H) 2.84–2.91 (m, 2 H) 2.92–2.96 (m, 2 H) 3.04 (s, 3 H) 3.37–3.47 (m, 4 H) 3.59 (t, *J* = 7.6 Hz, 2 H) 4.84 (s, 2 H) 4.92 (br s, 2 H) 7.32–7.36 (m, 4 H) 7.46 (d, *J* = 8.0 Hz, 2 H) 7.55 (d, *J* = 7.8 Hz, 2 H) 7.60 (s, 1 H) 7.68 (s, 1 H) 10.95 (s, 1 H). ^13^C NMR (125 MHz, DMSO-*d*_6_) δ ppm 18.72, 21.06, 24.82, 25.25, 41.95, 46.08, 46.26, 58.04, 59.17, 59.22, 113.83, 129.47, 129.60, 130.49, 131.00, 131.48, 131.53, 131.69, 131.74, 135.88, 136.28, 139.59, 139.77, 148.13, 167.51, 186.57. MS (FD) *m*/*z* found: 486.2744 (M+), 487.2778 (M + H), Calc: 486.2751.

**(7)**  **(3~{E},5~{E})-3,5-bis[(4-methoxyphenyl)methylene]-1-[3-(4-hydroximino-1-piperidyl)propanoyl]piperidin-4-one methiodide (4g)**

Yield 81%. MP: 179.5 °C. ^1^H NMR (500 MHz, DMSO-*d*_6_) δ ppm 2.57–2.68 (m, 2 H) 2.87–2.94 (m, 2 H) 2.97 (t, *J* = 7.5 Hz, 2 H) 3.04 (s, 3 H) 3.37–3.51 (m, 4 H) 3.61 (t, *J* = 7.5 Hz, 2 H) 3.85 (s, 3 H) 3.84 (s, 3 H) 4.84 (br s, 2 H) 4.92 (br s, 2 H) 7.09 (t, *J* = 7.5 Hz, 4 H) 7.54 (d, *J* = 8.5 Hz, 2 H) 7.62 (s, 1 H) 7.59 (s, 1 H) 7.66 (s, 1 H) 7.64 (s, 1 H) 10.92–10.98 (m, 1 H). ^13^C NMR (125 MHz, DMSO-*d*_6_) δ ppm 18.74, 24.84, 25.32, 41.90, 46.13, 46.32, 55.41, 55.47, 58.09, 59.21, 59.25, 113.38, 114.40, 114.50, 126.87, 130.27, 130.39, 132.42, 133.03, 135.56, 136.02, 148.14, MS (FD) *m*/*z* found: 518.2676 (M+), 519.2713 (M + H), Calc: 518.2649.

**(8)**  **(3~{E},5~{E})-1-[3-(4-hydroximino-1-piperidyl)propanoyl]-3,5-bis[(3,4,5-trimethoxyphenyl)methylene]piperidin-4-one methiodide (4h)**

Yield 79%. MP: 207 °C. ^1^H NMR (500 MHz, DMSO-*d*_6_) δ ppm 2.53–2.62 (m, 2 H) 2.62–2.70 (m, 2 H) 2.86–2.92 (m, 2 H) 2.99 (t, *J* = 7.4 Hz, 2 H) 3.03 (s, 3 H) 3.39–3.48 (m, 2 H) 3.62 (t, *J* = 7.5 Hz, 2 H) 3.74 (s, 6 H) 3.84 (s, 6 H) 3.88 (s, 6 H) 4.89 (s, 2 H) 4.98 (br s, 2 H) 6.91 (s, 2 H) 6.94 (s, 2 H) 7.62 (s, 1 H) 7.68 (s, 1 H) 10.95 (s, 1 H). ^13^C NMR (125 MHz, DMSO-*d*_6_) δ ppm 18.73, 24.82, 25.29, 46.30, 56.13, 58.12, 58.98, 59.22, 60.19, 108.03, 108.49, 113.56, 129.75, 129.82, 131.67, 138.86, 148.12, 152.91,167.75, 186.42. MS (FD) *m*/*z* found: 638.3079 (M+), 639.3112 (M + H), Calc: 638.3072.

### 5.2. Statistical Analyses

The physicochemical constants used in the QSAR studies were taken from the literature [22]. In the case of the MR values, the figure for a hydrogen atom was 1.03, not 0.00. The enones **3h** and **4h** had substituents in three locations in the arylidene aryl rings. Hence, to compare the sizes of substituents in all members of series **3** and **4,** the figure of 2.06 (2 × 1.03) was added to the MR constants for monosubstituted compounds while 1.03 was added to the MR values of the aryl groups in the disubstituted compounds **3d** and **4d**. The linear and semilogarithmic plots were made using a commercial package (Release 17.0, Chicago, IL, USA, 2008) [34]. 

### 5.3. Cytotoxicity Assays

A literature procedure was followed when the compounds in series **3** and **4** were evaluated against three human oral squamous cell carcinoma cell lines Ca9-22 (RCB-1976), HSC-2 (RCB1945), HSC-4 (RCB1902) (purchased from RIKEN Cell Bank, Tukuba, Japan) and three human normal oral cells, gingival fibroblast (HGF), periodontal ligament fibroblast (HPLF) and pulp cells (HPC) (established according to the guideline of the intramural ethic committee, A0808) [35] except the duration of incubation was increased from 24 h to 48 h [36]. In brief, various concentrations of the compounds were added to the DMEM media, which was supplemented by 10% heat-inactivated fetal bovine serum. Incubation was undertaken at 37 °C. At the completion of the experiment, cell viability was determined by the MTT method [36].

The method used for the evaluation of various compounds against human CEM cells has been reported previously [6]. In brief, cells were grown in RPMI-1640 medium supplemented with 10% heat-inactivated fetal bovine serum. Different concentrations of compounds were added to the media, and after 24 h incubation, cytotoxicity was measured by the MTT method.

### 5.4. Effect on Mitochondrial Function

The effect of **4b**,**c** on the mitochondrial membrane potential of CEM cells was determined using a literature method [6]. In brief, the cells were incubated with the compounds for 8 h. The neoplasms were then stained with the JC-1 dye and the results were obtained using flow cytometry.

### 5.5. Cell Cycle Analysis

Treated and untreated Ca9-22 cells (approximately 106 cells) were harvested, fixed with 1% paraformaldehyde, treated with 0.2 mg/mL RNase A (preheated for 10 min at 100 °C to inactivate DNase), stained for 15 min with 0.01% propidium iodide in the presence of 0.01% NP-40 to prevent cell aggregation, filtered through Falcon^®^ cell strainers (40 μM) (Corning, NY, USA), subjected to cell sorting (SH800 Series; SONY Imaging Products and Solutions Inc., Kanagawa, Japan) and then analyzed with Cell Sorter Software version 2.1.2. (SONY Imaging Products and Solutions Inc., Kanagawa, Japan), as described previously [37].

## Figures and Tables

**Table 1:** Evaluation of **3a**–**i** and **4a**–**h** against Ca9-22, HSC-2 and HSC-4 neoplastic cells.

Compound	Ca9-22		HSC-2		HSC-4		Average	
	CC_50_ (µM) ^a^	SI ^b^	CC_50_ (µM) ^a^	SI ^b^	CC_50_ (µM) ^a^	SI ^b^	CC_50_ (µM) ^a^	SI ^b^
**3a**	0.65 ± 0.07	16.9	0.89 ± 0.06	12.4	0.44 ± 0.16	25.0	0.66	18.1
**3b**	0.37 ± 0.19	28.1	0.73 ± 0.13	14.3	0.20 ± 0.08	52.0	0.43	31.5
**3c**	0.12 ± 0.08	113	0.66 ± 0.14	20.5	0.18 ± 0.03	75.0	0.32	69.5
**3d**	0.02 ± 0.01	180	0.10 ± 0.02	35.9	0.07 ± 0.00	51.3	0.06	89.1
**3e**	0.03 ± 0.00	109	0.12 ± 0.09	27.3	0.09 ± 0.02	36.3	0.08	57.5
**3f**	0.47 ± 0.04	15.3	0.75 ± 0.04	9.57	0.43 ± 0.12	16.7	0.55	13.9
**3g**	0.59 ± 0.06	18.3	1.56 ± 0.55	6.92	0.65 ± 0.02	16.6	0.93	13.9
**3h**	0.07 ± 0.03	43.9	0.23 ± 0.02	13.4	0.30 ± 0.15	10.2	0.20	22.5
**3i**	16.5 ± 2.35	5.10	18.0 ± 2.06	4.68	19.3 ± 1.61	4.36	17.9	4.71
**4a**	0.56 ± 0.01	30.4	1.45 ± 0.16	11.7	0.58 ± 0.04	29.3	0.86	23.8
**4b**	0.29 ± 0.23	28.7	0.42 ± 0.14	19.8	0.21 ± 0.01	39.6	0.31	29.4
**4c**	0.02 ± 0.01	202	0.06 ± 0.01	67.3	0.09 ± 0.03	44.9	0.06	105
**4d**	0.01 ± 0.00	236	0.03 ± 0.00	78.7	0.03 ± 0.00	78.7	0.02	131
**4e**	0.02 ± 0.00	104	0.05 ± 0.01	41.4	0.06 ± 0.01	34.5	0.04	60.0
**4f**	0.18 ± 0.02	53.1	0.46 ± 0.10	20.8	0.40 ± 0.23	23.9	0.35	32.6
**4g**	0.31 ± 0.17	41.0	0.68 ± 0.04	18.7	0.66 ± 0.05	19.2	0.55	26.3
**4h**	0.07 ± 0.01	20.4	0.22 ± 0.04	6.50	0.18 ± 0.04	7.94	0.16	11.6
Melphalan	48.5 ± 1.33	>3.79	14.4 ± 2.08	>12.8	17.4 ± 0.88	>10.6	26.7	>9.06
Doxorubicin	0.47 ± 0.12	15.6	0.11 ± 0.01	66.5	0.15 ± 0.05	48.7	0.24	43.6

^a^ The CC_50_ values are the concentrations of compounds required to kill 50% of the cells. ^b^ The letters SI refers to the selectivity index. The SI figures are the ratios of the average CC_50_ value of the compounds towards non-malignant HGF, HPLF and HPC cells (Table 2) and the CC_50_ figure of a compound against a specific neoplastic cell line.

**Table 2:** Evaluation of **3a**–**i** and **4a**–**h** against HGF, HPLF and HPC non-malignant cells.

Compound	CC_50_ (µM) ^a^				PSE ^b^
	HGF	HPLF	HPC	Average	
**3a**	7.11 ± 0.47	14.3 ± 3.79	11.5 ± 2.57	11.0	2742
**3b**	7.12 ± 0.04	9.73 ± 0.23	14.2 ± 2	10.4	7326
**3c**	6.40 ± 0.35	8.03 ± 0.06	26.1 ± 35.9	13.5	21,719
**3d**	1.91 ± 0.22	3.90 ± 1.42	4.97 ± 0.89	3.59	148,500
**3e**	1.67 ± 0.31	3.06 ± 0.16	5.07 ± 0.21	3.27	71,875
**3f**	5.70 ± 0.65	8.00 ± 0.20	7.85 ± 7.10	7.18	2527
**3g**	6.63 ± 0.06	12.1 ± 1.79	13.6 ± 0.60	10.8	1495
**3h**	2.18 ± 0.23	5.22 ± 0.69	1.80 ± 0.60	3.07	11,250
**3i**	64.9 ± 5.66	88.3 ± 4.56	99.3 ± 1.16	84.2	26.3
**4a**	9.65 ± 1.23	24.6 ± 1.10	16.8 ± 0.68	17.0	2767
**4b**	4.50 ± 1.39	9.13 ± 0.48	11.3 ± 2.59	8.31	9484
**4c**	1.65 ± 0.17	4.17 ± 1.44	6.30 ± 0.70	4.04	175,000
**4d**	1.15 ± 0.41	2.12 ± 0.20	3.81 ± 0.02	2.36	655,000
**4e**	1.15 ± 0.35	2.59 ± 0.27	2.46 ± 1.23	2.07	150,000
**4f**	5.12 ± 0.24	8.37 ± 0.81	15.2 ± 1.90	9.56	9314
**4g**	6.30 ± 0.27	16.2 ± 4.05	15.6 ± 3.16	12.7	4782
**4h**	0.62 ± 0.06	1.32 ± 0.04	2.35 ± 0.82	1.43	7250
Melphalan	157 ± 38.2	195 ± 4.04	>200	184	>33.9
Doxorubicin	3.44 ± 0.53	9.73 ± 0.47	8.77 ± 2.14	7.31	18,167

^a^ The CC_50_ values are the concentrations of the compounds required to kill 50% of the cells. ^b^ The letters PSE refer to the potency–selectivity expression. These figures are the product of the reciprocal of the average CC_50_ values against Ca9-22, HSC-2 and HSC-4 cells and the average SI value multiplied by 100.

**Table 3:** Evaluation of **3a–i** and **4a–h** against human CEM cells.

Compound	IC_50_ (µM) ^a^	Compound	IC_50_ (µM) ^a^
**3a**	5.39 ± 0.26	**4a**	7.62 ± 1.44
**3b**	1.78 ± 0.21	**4b**	1.05 ± 0.38
**3c**	15.9 ± 0.94	**4c**	4.96 ± 0.28
**3d**	8.49 ± 0.34	**4d**	5.19 ± 0.66
**3e**	11.6 ± 0.31	**4e**	0.87 ± 0.02
**3f**	8.42 ± 1.10	**4f**	4.18 ± 0.65
**3g**	4.35 ± 0.20	**4g**	13.0 ± 0.95
**3h**	2.31 ± 0.30	**4h**	5.12 ± 0.14
**3i**	11.1 ± 1.01		

^a^ The IC_50_ values are the concentrations of the compounds required to inhibit the cell growth by 50%.

**Table 4:** Evaluation of **3a**–**h** against certain human leukemia cells.

Compound	Leukemia Cells, IC_50_ (µM)					
	HL-60 (TB)	K-562	Molt-4	RPMI-8226	SR	Average
**3a**	2.60	0.40	0.69	0.32	0.33	0.85
**3b**	1.55	0.35	0.33	0.29	0.25	0.52
**3c**	0.56	0.16	0.28	0.16	0.04	0.24
**3d**	1.00	0.25	0.32	0.23	0.10	0.37
**3e**	1.55	0.28	0.40	0.30	0.10	0.50
**3f**	1.70	0.32	0.37	0.30	0.31	0.55
**3g**	2.82	1.20	2.95	0.65	0.62	1.88
**3h**	0.27	0.32	0.29	0.32	0.30	0.30

**Table 5:** Evaluation of **3a**–**h** against certain human colon cancer cells.

Compound	All Cell Lines,	Colon Cancer Cells, IC_50_ (µM)							
	IC_50_ (µM)	Colo205	HCC-2998	HCT-116	HCT-15	HT29	KM12	SW-620	Average
**3a**	1.51	1.70	1.74	0.68	1.38	1.20	0.66	0.32	1.10
**3b**	1.15	1.50	1.41	0.22	0.37	0.32	0.23	0.20	0.61
**3c**	1.62	0.35	0.31	0.21	0.27	0.30	0.22	0.15	0.26
**3d**	1.30	0.39	0.36	0.32	0.27	0.32	0.25	0.23	0.31
**3e**	1.79	0.40	1.17	0.49	0.36	0.33	0.29	0.20	0.46
**3f**	1.23	1.15	1.58	0.36	0.55	0.35	0.28	0.29	0.65
**3g**	1.28	2.04	1.86	1.74	2.14	2.04	1.74	1.38	1.84
**3h**	1.22	0.26	0.21	0.21	1.23	0.33	0.20	0.30	0.39

**Table 6:** Evaluation of **3c**, **d**, **4c–e** for certain drug-like properties and oral bioavailability ^a^.

Compound	MW (g/mol)	log P	HBA	HBD	RB	PSA (Å^2^)	Oral Bioavailability Score
**3c**	512.43	4.31	5	1	6	73.21	0.56
**3d**	581.32	5.37	5	1	6	73.21	0.17
**4c**	654.37	2.67	4	1	6	69.97	0.56
**4d**	723.26	3.70	4	1	6	69.97	0.56
**4e**	675.47	0.88	8	1	8	161.61	0.17
Ideal compound	≯500	≯5	≯10	≯5	<10	<140Å^2^	>0.50

^a^ The abbreviations in some of the headings of each column refer to molecular weight (MW), the logarithm of the partition coefficient (log P), hydrogen bond acceptor atoms (HBA), hydrogen bond donor atoms (HBD), rotatable bonds (RB) and polar surface area (PSA).

**Table 7:** The two most potent compounds in Table 1 and Table 3, Table 4, Table 5 (series **3**) and Table 1 and Table 3 (series **4**).

Table	Series 3	Series 4
1	d, e	d, e
3	b, h	b, e
4	c, h	--
5	c, d	--

## Data Availability

Not applicable.

## Supplementary Materials

The following are available online, Table S1. Correlation between some physicochemical constants and cytotoxic properties. Table S2. Correlations between some physicochemical constants and selectivity index (SI) values.

## Sample Availability

Samples of the compounds are available from the corresponding authors.
