# Supplementary material for: Design, Synthesis and Tumour-Selective Toxicity of Novel 1-[3-{3,5-Bis(benzylidene)-4-oxo-1-piperidino}-3-oxopropyl]-4-piperidone Oximes and Related Quaternary Ammonium Salts
Source: Molecules. 2021 Nov 25;26(23):7132. doi: 10.3390/molecules26237132 (PMC8659243; doi:10.3390/molecules26237132)
Supplement: Supplementary file 1 [file molecules-26-07132-s001.zip › molecules-1444661-supplementary.pdf]

SUPPLEMENTARY SECTION

Design, Synthesis and Tumour-Selective Toxicity of Novel 1-[3-{3,5-Bis(benzylidene)-4-oxo-1-piperidino}-3-oxopropyl]-4-piperidone Oximes and Related Quaternary Ammonium Salts

Praveen K. Roayapalley [1,*], Jonathan R. Dimmock [1], Lisett Contreras [2], Karol S. Balderrama [2], Renato J. Aguilera [2], Hiroshi Sakagami [3], Shigeru Amano [3], Rajendra K. Sharma [4] and Umashankar Das [1]

[1] Drug Discovery and Development Research Cluster, University of Saskatchewan, Saskatoon, Saskatchewan S7N 5E5, Canada; jr.dimmock@usask.ca (J.R.D.); umashankar.usask@gmail.com (U.D.)

[2] Department of Biological Sciences and Border Biomedical Research Center, the University of Texas at El Paso, El Paso, TX 79968-0519, USA; lcontreras4@miners.utep.edu (L.C.); karolsbalderrama@gmail.com (K.B.); raguilera@utep.edu (R.J.A.)

[3] Meikai University Research Institute of Odontology, Sakado, Saitama 350-0283, Japan; sakagami@dent.meikai.ac.jp (H.S.) shigerua@dent.maikai.a.jp (S.A.)

[4] Department of Pathology and Laboratory Medicine, College of Medicine, University of Saskatchewan,
Saskatoon, Saskatchewan S7N 5E5, Canada; rajendra.sharma@usask.ca

* Correspondence: rpraveen.sp@usask.ca

Table S1. Correlation between some physicochemical constants and cytotoxic properties……………S2

Table S2. Correlations between some physicochemical constants and selectivity index (SI) values…S3

Table S1. Correlations between some physicochemical constants and cytotoxic potencies.

Compounds	Cell line	Physicochemical parameter	Plot[a]	p value	Correlation[b]
**3a-i**	Ca9-22	σ	sl	<0.01	-
**3a-i**	HSC-2	σ	sl	<0.01	-
**3a-i**	HSC-4	σ	sl	<0.05	-
**3a-i**	HSC-4	π	sl	<0.1	-
**3a-h**	Ca9-22	σ	l	<0.05	-
**3a-h**	Ca9-22	σ	sl	<0.01	-
**3a-h**	HSC-2	σ	l	<0.05	-
**3a-h**	HSC-2	σ	sl	<0.01	-
**3a-h**	HSC-4	σ	l	<0.01	-
**3a-h**	HSC-4	σ	sl	<0.01	-
**4a-h**	Ca9-22	σ	sl	<0.05	-
**4a-h**	HSC-2	σ	sl	<0.05	-
**4a-h**	HSC-4	σ	l	<0.05	-
**4a-h**	HSC-4	σ	sl	<0.05	-

[a] The plots made are linear (l) and semilogarithmic (sl).

[b] In all cases correlations are negative (-).

Table S2. Correlations between some physicochemical constants and selectivity index (SI) values.

Compounds	Cell line	Physicochemical parameter	Plot[a]	p value	Correlation[b]
**3a-i**	Ca9-22	σ	l	<0.01	+
**3a-i**	Ca9-22	σ	sl	<0.01	+
**3a-i**	Ca9-22	π	l	<0.05	+
**3a-i**	Ca9-22	π	sl	<0.1	+
**3a-i**	HSC-2	σ	l	<0.01	+
**3a-i**	HSC-2	σ	sl	<0.01	+
**3a-i**	HSC-2	π	l	<0.1	+
**3a-i**	HSC-2	π	sl	<0.1	+
**3a-i**	HSC-4	σ	sl	<0.05	+
**3a-i**	HSC-4	π	sl	<0.1	+
**3a-h**	Ca9-22	σ	l	<0.01	+
**3a-h**	Ca9-22	σ	sl	<0.01	-
**3a-h**	HSC-2	σ	l	<0.1	+
**3a-h**	HSC-2	σ	sl	<0.01	+
**4a-h**	Ca9-22	σ	sl	<0.1	+
**4a-h**	Ca9-22	π	l	<0.05	+
**4a-h**	Ca9-22	π	sl	<0.1	+
**4a-h**	HSC-2	σ	l	<0.1	+
**4a-h**	HSC-2	σ	sl	<0.05	-
**4a-h**	HSC-2	π	l	<0.05	+
**4a-h**	HSC-4	σ	sl	<0.01	-
**4a-h**	HSC-4	π	l	<0.05	+

[a] The plots made are linear (l) and semilogarithmic (sl).

[b] Correlations are either positive (+) or negative (-).
